# The role of m^6^A RNA methylation in human cancer

**DOI:** 10.1186/s12943-019-1033-z

**Published:** 2019-05-29

**Authors:** Xiao-Yu Chen, Jing Zhang, Jin-Shui Zhu

**Affiliations:** Department of Gastroenterology, Shanghai Jiao Tong University Affiliated Sixth People’s Hospital, Yishan Road 600, Shanghai, 200233 China

**Keywords:** N^6^-methyladenosine, Cancer, RNA methylation, Prognosis, Growth, Metastasis

## Abstract

N^6^-methyladenosine (m^6^A) is identified as the most common, abundant and conserved internal transcriptional modification, especially within eukaryotic messenger RNAs (mRNAs). M^6^A modification is installed by the m^6^A methyltransferases (METTL3/14, WTAP, RBM15/15B and KIAA1429, termed as “writers”), reverted by the demethylases (FTO and ALKBH5, termed as “erasers”) and recognized by m^6^A binding proteins (YTHDF1/2/3, IGF2BP1 and HNRNPA2B1, termed as “readers”). Acumulating evidence shows that, m^6^A RNA methylation has an outsize effect on RNA production/metabolism and participates in the pathogenesis of multiple diseases including cancers. Until now, the molecular mechanisms underlying m^6^A RNA methylation in various tumors have not been comprehensively clarified. In this review, we mainly summarize the recent advances in biological function of m^6^A modifications in human cancer and discuss the potential therapeutic strategies.

## Introduction

According to MODOMICS, 163 different chemical modifications in RNA have been identified in all living organisms by the end of 2017 [1]. Among these modifications, N^6^-methyladenosine (m^6^A), methylated at the N^6^ position of adenosine, has been considered as the most pervasive, abundant and conserved internal transcriptional modification within eukaryotic messenger RNAs (mRNAs) [2], microRNAs (miRNAs) [3] and long non-coding RNAs (lncRNAs) [4]. RNA m^6^A is enriched near stop codon and 3′ untranslated terminal region (UTR) [5, 6] and translated near 5′ UTR in a cap-independent manner [7], thereby affecting RNA transcription, processing, translation and metabolism.

**Fig. 1:**
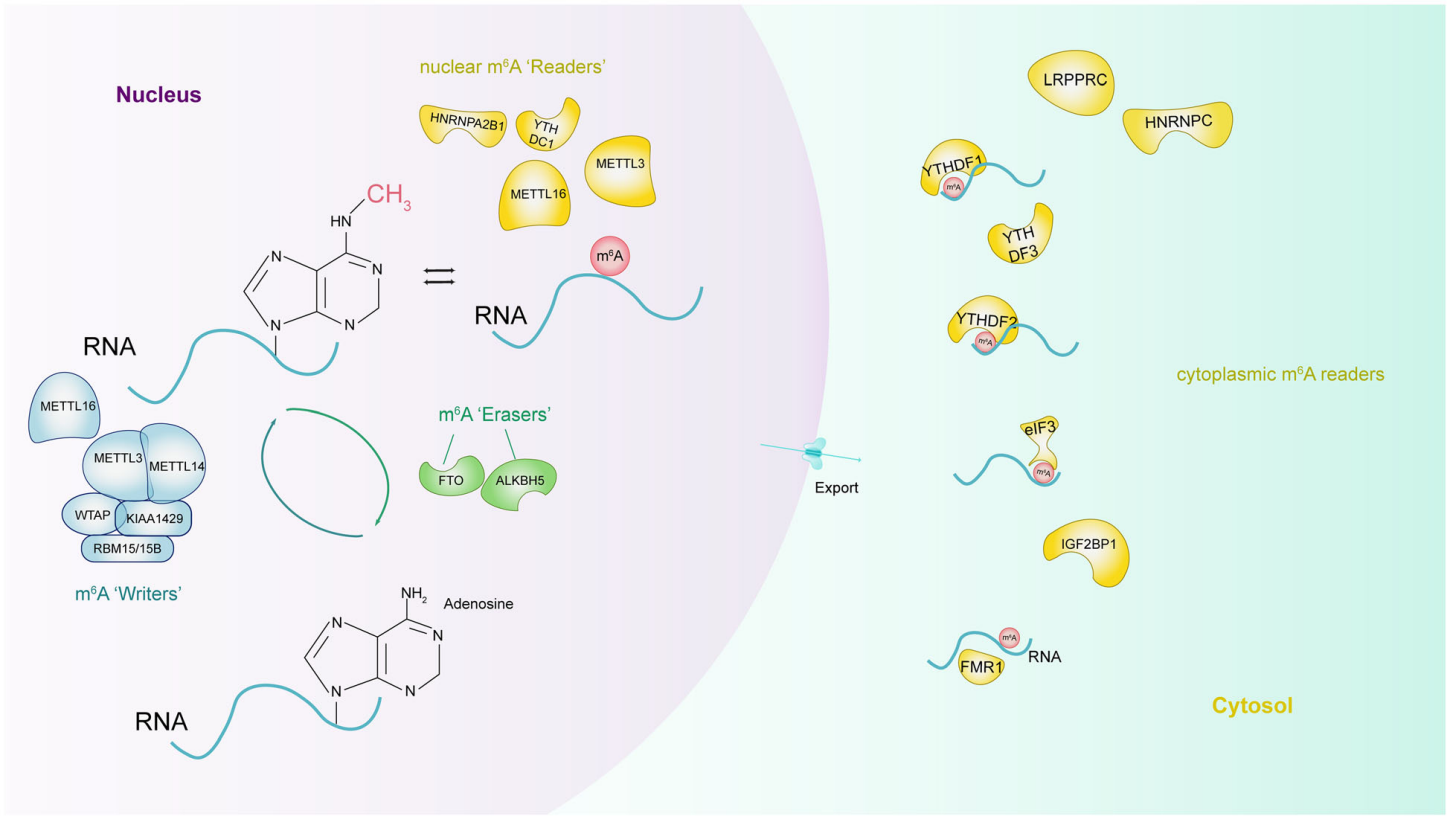
Molecular composition of m^6^A RNA methylation. M^6^A methylation is a dynamic and reversible process coordinated by a series of methyltransferases (METTL3/14, WTAP, RBM15/15B, and KIAA1429, termed as “m^6^A writers”), demethylases (FTO and ALKBH5, “m^6^A erasers”) and identifiers (YTHDF1/2/3, YTHDC1, HNRNPA2B1, HNRNPC, eIF3, FMR1, and LRPPRC, “m^6^A. ‘Readers”)

The deposition of m^6^A is encoded by a methyltransferase complex involving three homologous factors jargonized as ‘writers’, ‘erasers’ and ‘readers’ (Fig. 1). Methyltransferase-like 3 (METTL3) [8], METTL14 [9], Wilms tumor 1-associated protein (WTAP) [10], RBM15/15B [11] and KIAA1429 [12] are categorized as the components of ‘writers’ that catalyze the formation of m^6^A; ‘erasers’, fat mass and obesity-associated protein (FTO) [13] and alkB homologue 5 (ALKBH5) [14], selectively remove the methyl code from target mRNAs; ‘Readers’ are capable of decoding m^6^A methylation and generating a functional signal, including YT521-B homology (YTH) domain-containing protein [15], eukaryotic initiation factor (eIF) 3 [11], IGF2 mRNA binding proteins (IGF2BP) families [16] and heterogeneous nuclear ribonucleoprotein (HNRNP) protein families [17]. YTH domain can recognize m^6^A through a conserved aromatic cage [18] and another two proteins FMR1, LRPPRC “read” this modification [19, 20]. Contrary to the conventional ‘writer’-‘eraser’-‘reader’ paradigm, few studies reveal METTL3/16 as a m^6^A ‘writer’ or ‘reader’ [21].

M^6^A RNA modification is a dynamic and reversible process which was corroborated by the discovery of ‘eraser’ in 2011 [13]. It is associated with multiple diseases such as obesity, infertility and cancer [22]. In this review, we summarize the function and therapeutic advances of m^6^A modifications in human cancer and provide their promising applications in the treatment of these malignant tumors (Table 1).

**Table 1:** Multiple functions exerted by m^6^A RNA methylation in various diseases

Disease	Related targets	M^6^A component	Function	Role in diseases	Source of experimental evidence	Regulation	Year	Refs
AML	c-MYC, BCL2, PTEN	METTL3	Writers	oncogene	HSPCs, AML MOLM-13 cells	Up-regulation	2017	[70]
AML	CEBPZ	METTL3	Writers	oncogene	AML cells, immunodeficient mice	Up-regulation	2017	[23]
AML	MYB, MYC	METTL14	Writers	oncogene	AML cell lines	Up-regulation	2017	[68]
AML	mTOR	WTAP	Writers	oncogene	511 newly diagnosed AML patient samples, Ba/F3 cell line, AML cell lines	Down-regulation	2014	[69]
AML	ASB2, RARA	FTO	Erasers	oncogene	MONOMAC-6 and NB4 cells	Down-regulation	2017	[67]
AML	MYC, CEBPA	FTO	Erasers	oncogene	27 human leukemia cell lines	Up-regulation	2018	[24]
AML	Tal1…	YTHDF2	Readers	anti- oncogene	HSPCs, mouse…	Down-regulation	2018	[72]
Bladder cancer	AFF4, MYC	METTL3	Writers	oncogene	Bladder cancer cell lines, mouse bladder cancer samples	Up-regulation	2019	[44]
Breast cancer	HBXIP	METTL3	Writers	oncogene	24 clinical tumor samples, MCF-7, MDA-MB-468 cells	Up-regulation	2018	[78]
Breast cancer	NANOG	ALKBH5	Erasers	oncogene	BCSCs	Up-regulation	2016	[79]
CRC	WT1, TBL1	WTAP	Writers	oncogene	115 patient samples with CRC, colon cancer cell lines	Down-regulation	2016	[81]
Endometrial cancer	AKT…	METTL3/14	Writers	anti- oncogene	Cancer samples, endometrial cancer cell lines, mice	Down-regulation	2018	[66]
GBM	ADAM19	METTL3/14	Writers	anti- oncogene	GSC, GSC-grafted mice	Down-regulation	2017	[74]
GBM	FOXM1	ALKBH5	Erasers	oncogene	GSCs	Up-regulation	2017	[73]
HCC	SOCS2	METTL3	Writers	oncogene	MHCC97L, Huh-7 and HepG2 cell lines, BABL/cAnN-nude mice	Down-regulation	2017	[37]
HCC	miR-126	METTL14	Writers	anti-oncogene	HepG2 cell	Up-regulation	2017	[26]
HCC	miR-145	YTHDF2	Readers	oncogene	clinical tissue, HepG2 cell line	miR-145 suppresses YTHDF2	2017	[77]
HCC, AML…	SRF…	IGF2BP1	Readers	oncogene	HepG2, K562, hESCs cell lines…	Up-regulation	2019	[16]
impaired fertility	Uhrf1…	ALKBH5	Erasers	spermatogenesis	HeLa cells, mouse testicular cells	Up-regulation	2013	[14]
Lung cancer	EGFR, TAZ	METTL3	Writers	oncogene	Human lung cancer cell lines, HeLa, HEK293T cells	Up-regulation	2016	[46]
NPC	ZNF750, FGF14	METTL3	Writers	oncogene	NPC biopsy samples and cell lines	Down-regulation	2018	[45]
Obesity	SRSF2	FTO	Erasers	adipogenesis	3 T3-L1 cell	Decreased RNA binding ability	2014	[28]
Pancreatic cancer	RBM17…	METTL3	Writers	oncogene	Pancreatic cancer cell lines	Up-regulation	2018	[31]

*HSPCs* hematopoietic stem/progenitor cells, *AML* acute myeloid leukemia, *GBM* Glioblastoma, *GSC* glioblastoma stem cell, *HCC* hepatocellular cancer, *CRC* colorectal cancer, *BCSCs* Breast cancer stem cells, *NPC* Nasopharyngeal carcinoma

### Biological function of m^6^A modification in mammals

**Fig. 2:**
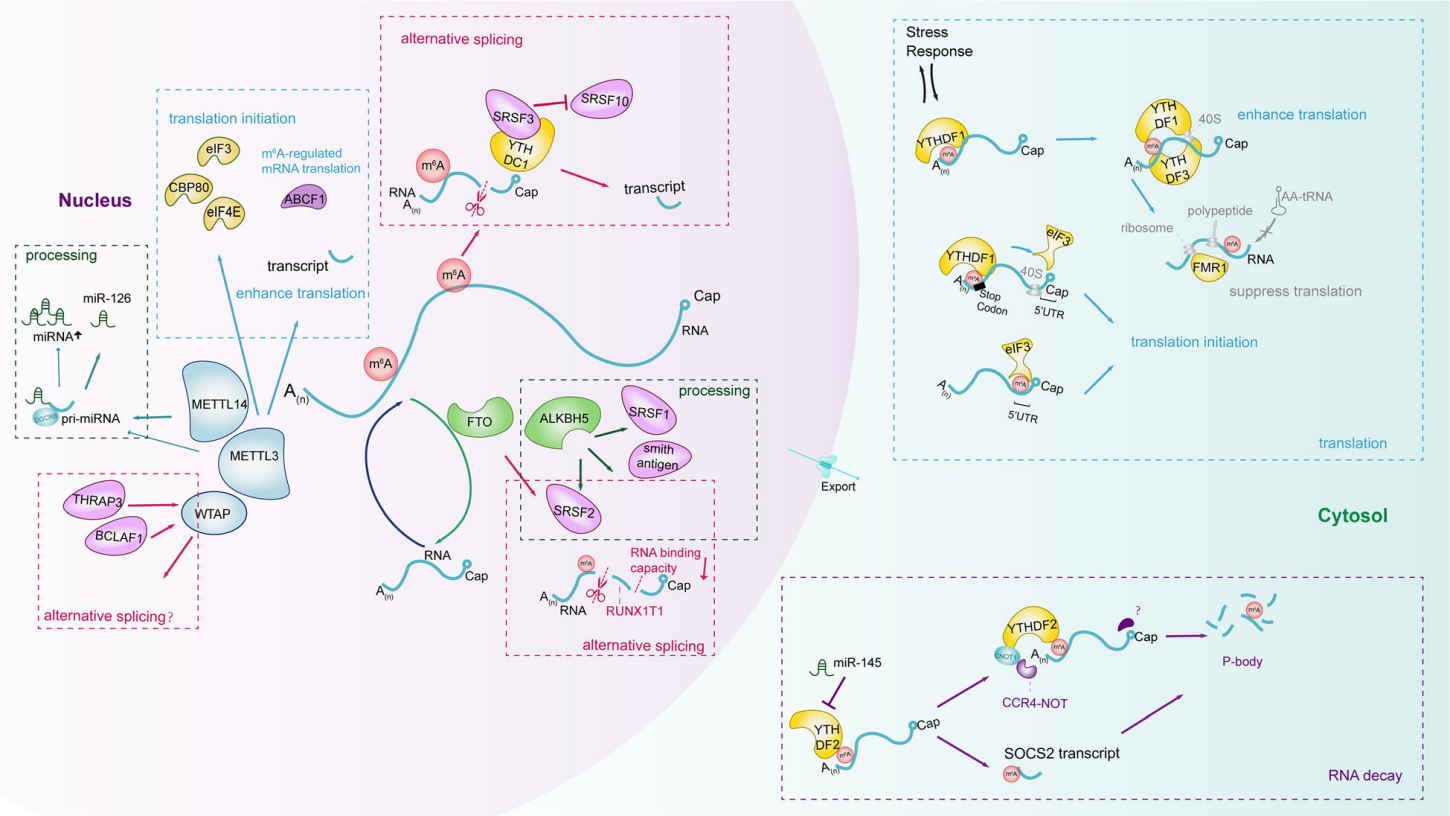
Regulatory Functions of m^6^A modification in RNA splicing, processing, translation and degradation. M^6^A RNA modification is involved in regulating the life cycle of RNA including RNA splicing (regulated by WTAP, FTO, ALKBH5 and YTHDC1), RNA processing (regulated by METTL3/14 and ALKBH5), RNA translation (regulated by METTL3, YTHDF1/3, eIF3 and FMR1) and RNA degradation (regulated by YTHDF2)

Recent years have witnessed a substantial progress of m^6^A post-transcriptional modification in regulating RNA transcription [23, 24], processing event [25–27], splicing [28–33], RNA stabilities [34–40] and translation [42–49] (Fig. 2).

#### M^6^A modification in RNA transcript

METTL3 and FTO are implicated in regulating transcription of CCAAT-enhancer binding protein (CEBP) family. METTL3 is localized to the starting sites of CEBPZ, which is required for recruitment of METTL3 to chromatin [23]. CEBPA is identified as an exclusive transcription factor displaying a positive correlation with FTO and regulating its transcription in acute myeloid leukemia (AML) [24].

#### M^6^A modification in RNA processing

M^6^A modifications promote the initiation of miRNA biogenesis [3] and regulate nuclear mRNA processing events [25]. METTL3 recognizes the pri-miRNAs by microprocessor protein DGCR8 and causes the elevation of mature miRNAs and concomitant reduction of unprocessed pri-miRNAs in breast cancer [3]. METTL14 interacts with DGCR8 to modulate pri-miR-126 and suppresses the metastatic potential of hepatocellular carcinoma (HCC) [26]. FTO can regulate poly(A) site and 3′ UTR length by interacting with METTL3 [25]. YTHDC1 knockout in oocytes exhibits massive defects and contributes to extensive alternative polyadenylation and 3′ UTR length alterations [27].

#### M^6^A modification in RNA splicing

M^6^A RNA modifications that overlap in space with the splicing enhancer regions affect alternative RNA splicing by acting as key pre-mRNA splicing regulators [28]. Inhibiton of m^6^A methyltransferase impacts gene expression and alternative splicing patterns [29]. FTO regulates nuclear mRNA alternative splicing by binding with SRSF2 [25]. FTO and ALKBH5 regulate m^6^A around splice sites to control the splicing of Runt-related transcription factor 1 (RUNX1T1) in exon [28], and removal of m^6^A by FTO reduces the recruitment of SRSF2 and prompts the skipping of exon 6, leading to a short isoform of RUNX1T1 [30]. Depletion of METTL3 is associated with RNA splicing in pancreatic cancer [31]. WTAP is enriched in some proteins involved in pre-mRNA splicing [32]. But, some studies show that, M^6^A is not enriched at the ends of alternatively spliced exons and METTL3 unaffects pre-mRNA splicing in embryonic stem cells [33].

#### M^6^A modification in RNA degradation

M^6^A is a determinant of cytoplasmic mRNA stability [34], and reduces mRNA stability [35]. A RNA decay monitoring system is adopted to investigate the effects of m^6^A modifications on RNA degradation [36]. Knockdown of METTL3 abolishes SOCS2 m^6^A modification and augments SOCS2 expression [37]. M^6^A-mediated SOCS2 degradation also relies on m^6^A ‘reader’ YTHDFs [37], which accelerate the decay of m^6^A-modified transcripts [38] or target mRNA [39]. Knockout of m^6^A methyltransferase attenuates YTHDF2 specific binding with target mRNAs and increases their stability [40]. M^6^A RNA methylation also controls T cell homeostasis by targeting the IL-7/STAT5/SOCS pathways [41] and decreases the stability of MYC/CEBPA transcripts [24].

#### M^6^A modification in RNA translation

M^6^A modifications occur in mRNA and noncoding RNA (ncRNAs) to regulate gene expression in its 5′ or 3′ UTR [7, 42]. METTL3 enhances mRNA translation [8], while depletion of METTL3 selectively inhibits mRNAs translation in 5′UTR [43] and reduces AFF4 and MYC translation in bladder cancer [44] but increase that of zinc finger protein 750 and fibroblast growth factor 14 in nasopharyngeal carcinoma [45].

M^6^A modifications facilitate the initiated translation through interacting with the initiation factors eIF3, CBP80 and eIF4E in an RNA-independent manner [46]. Heat-shock-induced translation of heat-shock protein 70 (HSP70) alters the transcriptome-wide distribution of m^6^A [7] and affects DNA repair [47]. ABCF1-sensitive transcripts largely overlaps with METTL3-modified mRNAs and are critical for m^6^A-regulated mRNA translation [43]. In addition, FMR1 binds to hundreds of mRNAs to negatively regulate their translation [20]. YTHDF1 facilitates the translation of m^6^A-modified mRNAs in protein-synthesis and YTHDF3 acts in the initial stage of m^6^A-driven translation from circular RNAs (circRNAs) [38, 48, 49].

### M^6^A RNA modification in metabolic and developmental diseases

The methyltransferases and demethylases of m^6^A are associated with a variety of diseases, such as obesity [13, 50], type 2 diabetes mellitus (T_2_DM) [51], growth retardation, developmental delay, facial dysmorphism [52]. Besides, m^6^A modification affects infertility [14], developmental arrest [22], neuronal disorder [53] and infectious diseases [54, 55].

#### M^6^A modification in metabolic and infectious diseases

M^6^A modification is involved in metabolic abnormalities in patients with T_2_DM and obesity [56]. FTO regulates the energy homeostasis and dopaminergic pathway through FTO-dependent m^6^A demethylation [50, 51], and it is ubiquitous in adipose and muscle tissues, influencing RUNX1T1 splicing in adipogenesis [28, 30]. METTL3/14 reduce the abundance of Hepatitis C virus replication, but FTO promotes its production through YTHDF proteins [54]. M^6^A is also identified as a conserved modulatory symbol across Flaviviridae genomes, including dengue, Zika virus and West Nile virus [55].

#### M^6^A modification in infertility

Deficiency of demethylase ALKBH5 leads to the aberrant spermatogenesis and apoptosis with impaired fertility in testes and striking changes in DNA methyltransferase 1 (Dnmt1) and ubiquitin-like with PHD and RING finger domains 1 (Uhrf1) [14]. YTHDF2 is required for maternal transcriptome during oocyte maturation [57]. YTHDC1/2 determine the germline development in mouse [58], and YTHDC1 is essential for spermatogonia in males and oocyte maturation in females [27].

#### M^6^A modification in nervous system development

M^6^A modification regulates the pace of cerebral cortex development [59] and m^6^A-regulated histone modifications enhances self-renewal of neural stem cells by METTL3/14 [60]. M^6^A has dual effects on delaying tempo of corticogenesis by two distinct pathways: increased cell-cycle length and decreased mRNA decay [59]. M^6^A depletion decreases the decay of radial glia cells associated with stem cell maintenance, neurogenesis and differentiation [61].

#### M^6^A modification in inflammation and metabolism-related cancer

Cacinogenesis is characterized by stepwise accumulation of genetic/epigenetic alterations of different proto-oncogenes and tumor-suppressor genes following other diseases including chronic inflammation and metabolic diseases. METTL3/14 and FTO influence Hepatitis C virus replication and production, and endogenous mediators of inflammatory responses (proinflammatory cytokines, reactive oxygen, et al) can promote genetic/epigenetic alterations [62]. FTO affects RUNX1T1 splicing in adipogenesis [28, 30], and RUNX1T1 is essential for pancreas development [63]. Transcription factor forkhead box protein O1 (FOXO1) as another direct substrate of FTO, regulates gluconeogenesis in liver [64] and promotes the growth of pancreatic ductal adenocarcinoma [65].

### M^6^A RNA modification in human cancer

**Fig. 3:**
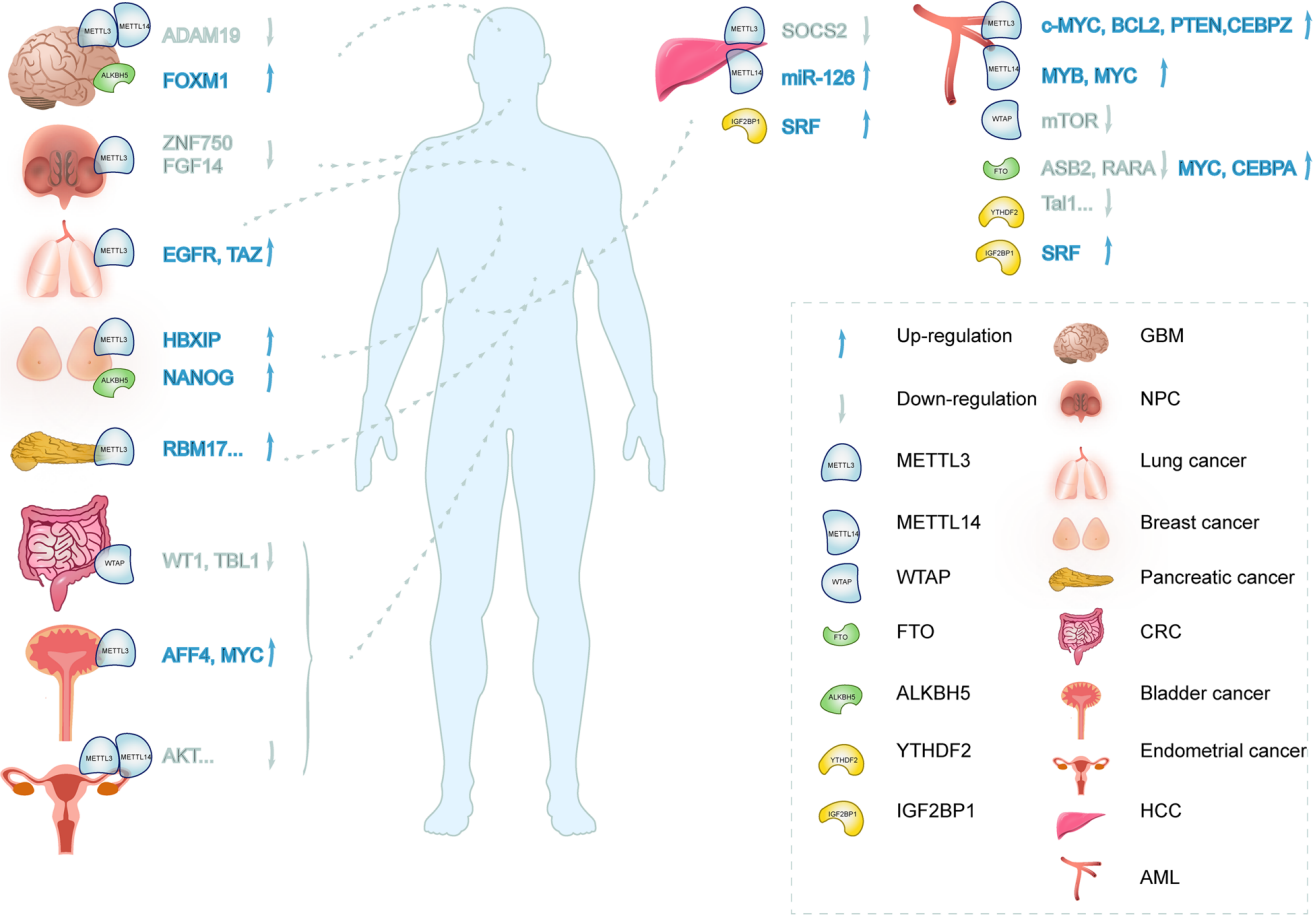
The role of m^6^A RNA modification in human cancer. M^6^A RNA modification is associated with the tumorigenesis of multiple malignancies including AML, GBM, HCC, CRC, NPC, breast cancer, lung cancer, pancreatic cancer, bladder cancer and endometrial cancer

Emerging evidence suggests that, m^6^A modification is associated with the tumor proliferation, differentiation, tumorigenesis [46], proliferation [66], invasion [46] and metastasis [26] and functions as oncogenes or anti-oncogenes in malignant tumors (Table 1 and Fig. 3).

#### Acute myeloid leukemia (AML)

FTO is highly expressed in AML with t(11q23)/MLL rearrangements, t(15;17)/PML-RARA, FLT3-ITD and/or NPM1 mutations and promotes leukemic cell transformation and tumorigenesis [67]. METTL3/14 are expressed in hematopoietic stem/progenitor cells (HSPCs) and AML cells with t(11q23), t(15;17), or t(8;21), control the terminal myeloid differentiation of HSPCs and promote the survival and proliferation of AML [68]. WTAP acts in cell proliferation and arrests the differentiation of leukemia [69].

M^6^A promotes the translation of *c-MYC*, *BCL2* and *PTEN* in AML [70]. METTL14 acts an oncogenic role by regulating its targets MYB/MYC through m^6^A modification [68]. YTHDF2, responsible for the decay of m^6^A-modified mRNA transcripts [40], is also associated with MYC in leukemia [71]. Besides, YTHDF2 stabilizes Tal1 mRNAs and increases its expansion in AML [72].

Collectively, these studies corroborate the functional importance of m^6^A modifications in leukemia, such as METTL3 [23, 70], METTL14 [68], FTO [24, 67] and YTHDF2 [24, 40] and they provide profound insights into development and maintenance of AML and self-renewal of leukemia stem/initiation cells through the downstream MYC and Tal1 pathways.

#### Glioblastoma (GBM)

METTL3/14 inhibit GSC growth, self-renewal and tumorigenesis, but FTO and ALKBH5 indicate poor survival in GBM by regulating ADAM19 and transcription factor FOXM1 [73, 74]. LncRNA antisense to FOXM1 (FOXM1-AS) promotes the interaction of ALKBH5 with FOXM1 nascent transcripts in the tumorigenesis of GSCs [73].

#### Lung cancer

M^6^A demethylase FTO is identified as a prognostic factor in lung squamous cell carcinoma (LUSC) and facilitates cell proliferation and invasion, but inhibits cell apoptosis by regulating MZF1 expression [75]. METTL3 acts as a oncogene in lung cancer by increasing EGFR and TAZ expression and promoting cell growth, survival and invasion [46]. METTL3-eIF3 caused mRNA circularization promotes the translation and oncogenesis of lung adenocarcinoma [46]. Besides, SUMOylation of METTL3 is of importance for the promotion of tumor growth at lysine residues K^177^, K^211^, K^212^ and K^215^ in non-small cell lung carcinoma (NSCLC) [76]. These studies provide insights into the critical roles of METTL3 and FTO in lung carcinoma.

#### Hepatocellular carcinoma (HCC)

METTL3 is related to a poor prognosis in HCC patients and promotes HCC cell proliferation, migration and colony formation by YTHDF2-dependent posttranscriptional silencing of SOCS2 [37]. But, METTL14 is an anti-metastatic factor and serves as a favorable factor in HCC by regulating m^6^A-dependent miRNA processing [26]. MiR-145 down-regulates YTHDF2 through targeting its mRNA 3′ UTR [77]. In conclusion, METTL3 upregulation or METTL14 downregulation predicts poor prognosis in patients with HCC and contributes to HCC progression and metastasis [26, 37]. METTL3 suppresses SOCS2 expression in HCC via the miR-145/m^6^A/YTHDF2 dependent axis [37, 77]. Thus, these studies suggest a new dimension of epigenetic alteration in liver carcinogenesis.

#### Breast cancer and colorectal cancer (CRC)

METTL3 is associated with the expression of mammalian hepatitis B X-interacting protein (HBXIP), displaying an aggressiveness in breast cancer. HBXIP-induced METTL3 promotes the proliferation of breast cancer via inhibiting tumor suppressor let-7 g [78]. Besides, ALKBH5 decreases the levels of m^6^A in NANOG mRNA and enhances its stability, leading to an increase of NANOG mRNA and protein levels in breast cancer stem cells (BCSCs) [79]. Another m^6^A eraser ‘FTO’ polymorphism has no association with the risk of CRC [80], but the m^6^A ‘writer’ WTAP is associated with carbonic anhydrase IV (CA4), which inhibits the proliferation and induces apoptosis and cycle arrest by repressing the Wnt signaling through targeting the WTAP-WT1-TBL1 axis [81].

#### Brief summary of m^6^A modification-related carcinogenesis

M^6^A RNA modifications regulate RNA production/metabolism and take part in the carcinogenesis. On the one hand, m^6^A-modified genes usually act a oncogenic role in cancer, leading to alterations of mRNA translation and acceleration of tumor progression, and decreasing m^6^A modification results in tumor development. On the other hand, given that SUMOylation of METTL3 represses its m^6^A methyltransferase capacity and results in tumor growth of NSCLC, modification of m^6^A methylase can determine the tumor development.

#### M^6^A modification in cancer treatment

M^6^A modification indicates new directions for the treatment of various cancers. Regulators or inhibitors of m^6^A modifications may provide the potential therapeutic strategies for cancers, such as MA2 in GBM [74], R-2HG/SPI1/FB23–2 in AML [24, 68, 82] and CA4 in CRC [81]. Meclofenamic acid (MA) as one of the selective FTO inhibitors is a non-steroidal anti-inflammatory drug by competing with FTO binding sites [83]. MA2, the ethyl ester derivative of MA, increases m^6^A modification, leading to the suppression of tumor progression [74, 83]. The expression of ASB2 and RARA is increased in hematopoiesis and they act as key regulators of ATRA-induced differentiation of leukemia cells [84]. FTO enhances the leukemogenesis of AML by inhibition of the ASB2 and RARA expression [67]. FB23–2, as another inhibitor of m^6^A demethylase FTO suppresses AML cell proliferation and promotes the cell differentiation and apoptosis [82].

ALKBH5 and FTO are α-ketoglutarate (α-KG)-dependent dioxygenases [85], which are competitively inhibited by D2-hydorxyglutarate (D2-HG) and elevated in isocitrate de-hydrogenases (IDH)-mutant cancers for transferring isocitrate to α-KG [86]. R-2-hydroxyglutarate (R-2HG), an metabolite by mutating IDH1/2 enzyme, exhibits anti-leukemia effects through increasing m^6^A levels in R-2HG-sensitive AML [24].

S-adenosylmethionine (SAM) serves as a cofactor substrate in METTL3/14 complex and its product S-adenosylhomocysteine (SAH) inhibits the methyltransferases by competing with adenosylmethionine [87]. 3-deazaadenosine (DAA) inhibits SAH hydrolase and interrupts insertion of m^6^A into mRNA substrates [88] and its analogs suppress the replication of various viruses editing m^6^A- mRNA in cancers [89, 90].

METTL14 acts an oncogenic role by regulating MYB/MYC axis through m^6^A modification [68]. SPI1, a hematopoietic transcription factor, directly inhibits METTL14 expression in malignant hematopoietic cells [68] and may be a potential therapeutic target for AML. CA4 inhibits the tumorigenicity of CRC by suppressing the WTAP-WT1-TBL1 axis [81].

#### Future prospect

M^6^A RNA modifications act by regulating RNA transcript, splicing, processing, translation and decay and participate in the tumorigenesis and metastasis of multiple malignancies. However, the underlying mechanisms of m^6^A modifications in cancer should be further addressed.. Besides FMR1 and LRPPRC, the function of ALKBH family in m^6^A RNA methylation is undetermined. METTL14 has different expression levels in various tumor tissues. Given a dual role of METTL14 either as a tumor suppressor [26] or an oncogene in cancer [68], its role in other cancers need be further elucidated. Though some inhibitors of m^6^A methylation have shown promising effects on cancer development [68, 81], novel therapeutic strategies for m^6^A RNA methylation should be further explored in the treatment of cancer.

## Data Availability

All data generated or analyzed during this study are included in this published article and its additional files.

## Abbreviations

ALKBH5: Alkb homologue 5
AML: Acute myeloid leukemia
BCLAF1: BCL2-associated transcription factor 1
BCSCs: Breast cancer stem cells
CA4: Carbonic anhydrase IV
CEBP: CCAAT-enhancer binding protein
circRNAs: Circular RNAs
CRC: Colorectal cancer
D2-HG: D2-hydorxyglutarate
DAA: 3-deazaadenosine
Dnmt1: DNA methyltransferase 1
eIF: eukaryotic initiation factor
FGF14: Fibroblast growth factor 14
FOXM1-AS: Antisense to FOXM1
FOXO1: Forkhead box protein O1
FTO: Fat mass and obesity-associated protein
GSCs: Glioblastoma stem-like cells
HBXIP: Hepatitis B X-interacting protein
HCC: Hepatocellular carcinoma
HNRNP: Heterogeneous nuclear ribonucleoprotein
HSP70: Heat-shock protein 70
HSPCs: Hematopoietic stem/progenitor cells
IDH: Isocitrate de-hydrogenases
IGF2BP: IGF2 mRNA binding proteins
lncRNAs: Long non-coding RNAs
LUSC: Lung squamous cell carcinoma
m^6^A: N^6^-methyladenosine
MA: Meclofenamic acid
METTL3: Methyltransferase-like 3
miRNAs: Micro RNAs
mRNAs: Messenger RNAs
ncRNAs: Noncoding RNAs
NSCLC: Non-small cell lung carcinoma
R-2HG: R-2-hydroxyglutarate
RUNX1T1: Runt-related transcription factor 1
SAH: S-adenosylhomocysteine
SAM: S-adenosylmethionine
SOCS2: Suppressor of cytokine signaling 2
SRSF: serine/arginine-rich splicing factor
T_2_DM: Type 2 diabetes mellitus
THRAP3: Thyroid hormone receptor-associated protein 3
Uhrf1: Ubiquitin-like with PHD and RING finger domains 1
UTR: Untranslated terminal region
WTAP: Wilms tumour 1-associated protein
YTHDF: YT521-B homology domain-containing protein family
ZNF750: Zinc finger protein 750
α-KG: Α-ketoglutarate
